# Antibiotic-Loaded Dendrimer Hydrogels in Periodontal Bone Regeneration: An In Vitro Release Feasibility Study

**DOI:** 10.3390/gels10090593

**Published:** 2024-09-14

**Authors:** Nicholas Yesbeck, Da Huang, Caroline Carrico, Parthasarathy Madurantakam, Hu Yang

**Affiliations:** 1 Department of Periodontics, Virginia Commonwealth University, Richmond, VA 23298, USA; nyesbeck@commonwealth-dentistry.com; 2 College of Biological Science and Engineering, Fuzhou University, Fuzhou 350108, China; huangda@fzu.edu.cn; 3 Dental Public Health and Policy, Virginia Commonwealth University School of Dentistry, Richmond, VA 23298, USA; ckcarrico@vcu.edu; 4 Department of General Practice, Virginia Commonwealth University School of Dentistry, Richmond, VA 23298, USA; 5 Linda and Bipin Doshi Department of Chemical and Biochemical Engineering, Missouri University of Science and Technology, Rolla, MO 65401, USA

**Keywords:** antibiotic, PAMAM dendrimer, hydrogel, cefazolin, local delivery

## Abstract

The prescription of a course of oral antibiotics following bone grafting procedures is a common practice in clinical periodontics to reduce surgical site infections. The goal of this study is to characterize the release profile of antibiotics via local delivery using dendrimer hydrogels (DH) and to analyze the effect of two different particulate bone allografts on the release of the antibiotics in vitro. DH were synthesized from polyamidoamine (PAMAM) dendrimer G5 and polyethylene glycol diacrylate, and cefazolin was chosen as the antibiotic. The antibiotic-loaded samples were bathed in PBS and incubated at 37 °C; aliquots were taken (1 h, 2 h, 3 h, 4 h, 5 h, 6 h, 12 h, 24 h, 48 h, 72 h) and analyzed using HPLC to determine the amounts of released cefazolin. In samples with DH, the estimated maximum concentration of cefazolin was 36.97 ± 2.39 μg/mL (95% CI: 34.58–39.36) with 50% released in 4.17 h (95%: 3.26–5.07) and an estimated growth rate of 0.27 (95% CI: 0.17–0.37). For samples without DH, the estimated maximum concentration of cefazolin was 167.4 ± 7.0 μg/mL (95% CI: 160.4–174.4) with 50% released in 2.36 h (95% CI: 2.05–2.67) and an estimated growth rate of 0.70 (95% CI: 0.54–0.87). We conclude that DH are a promising platform for sustained antibiotic release and that the presence of bone grafts did not significantly affect their release.

## 1. Introduction

Tooth loss can occur for several reasons, mostly due to caries, periodontal disease, trauma, or congenital absence. While various treatments to replace missing teeth exist, dental implants are the most popular option for such clinical situations. A dental implant is a titanium screw (in the form of a root of a tooth) that is surgically placed into the jawbone. Over a period of few weeks, the implants fuses to the jawbone by a process called osseointegration [1]. Today, over 3 million implants are placed annually in the USA, with the market projected to grow significantly by 2027 [2].

Diseased teeth are extracted from their housing in the jawbone by surgery. After tooth extraction, the bone at the extraction site undergoes permanent resorption in all dimensions [3,4]. Since adequate bone volume is a fundamental requirement for implant placement, efforts are directed to restore the volume of the bony socket using allogeneic bone grafts (processed bone from cadavers) [5,6]. However, these avascular grafts are susceptible to bacterial infection, with infection rates up to 12–17% [7,8], resulting in delayed healing or the need for graft removal [9,10,11]. Oral antibiotics are commonly prescribed when performing implant surgeries to prevent infections, although there is variation in the drug prescribed, the dose, and the duration [12,13,14,15]. Systemic antibiotics require high doses and patient compliance, cause adverse effects, and have poor tissue targeting, further limiting their efficacy [16,17].

**Figure 1:**
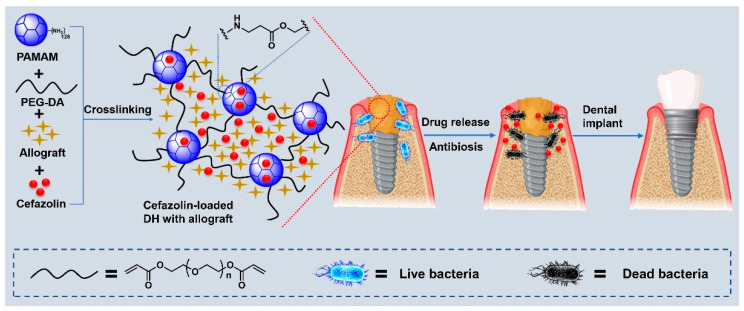
Schematic illustration of the preparation of cefazolin-loaded dendrimer hydrogels (DH) with allograft and their application in periodontal bone regeneration.

Local antibiotic delivery, in contrast, avoids systemic side effects, minimizes antibiotic resistance, and is available at the immediate site of interest. Various drug delivery systems, including liposomes, polymeric micelles, metal nanoparticles, silica nanoparticles, vesicles, and hydrogels have been developed for the controlled delivery of antibiotics [18]. Hydrogels render excellent biocompatibility and offer the capability to easily encapsulate hydrophilic drugs [19]. Dendrimer hydrogels (DHs) are a special class of hydrogels that are formed by linking together highly branched dendrimer macromolecules. Dendrimers are made up of layers of dendrons (i.e., concentric branching units) radiating from a central initiator core, where each layer is termed a generation (G). In general, the higher the generation of the dendrimer, the larger the molecule becomes, and the more end-group (termini) functionality it develops [20]. We used amine-terminated polyamidoamine (PAMAM) dendrimers to create a dendrimer hydrogel (DH) for cefazolin delivery, as PAMAM dendrimers are a well-established drug delivery system. Crosslinking PAMAM with polyethylene glycol diacrylate (PEG-DA) forms a hydrophilic degradable hydrogel with low cytotoxicity (Figure 1). We chose to study the PAMAM and PEG-based combination products because they are FDA-approved [21].

In this study, cefazolin, a first-generation cephalosporin, was chosen for local delivery because it is effective against the most common pathogens associated with allograft infections [22,23] and also the preferred antibiotic in penicillin-allergic patients [24]. Previous studies with local delivery of cefazolin showed prolonged surgical site concentrations above the minimum inhibitory concentration (MIC) when compared to intravenous administration [25].

The aim of the present study is to explore the potential of DH to deliver cephazolin intended for local delivery and the sustained release of antibiotics. We included two different bone allograft types: FDBA and DFDBA, to mimic the clinical practice of adding antibiotics to graft material and additionally evaluate whether the hydroxyapatite (in bone graft) increases the binding and retention of cephazolin. This study lays the foundation for future experiments in utilizing DH in oral bone regeneration.

## 2. Results and Discussion

**Figure 2:**
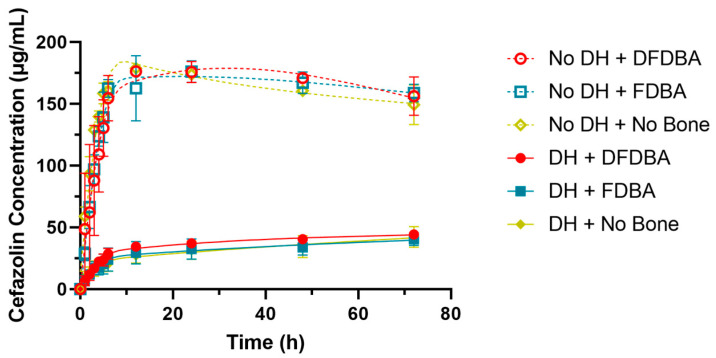
Restricted cubic spline model for cefazolin concentration by time, bone graft material, and presence of DH.

All splines in the RCS model were statistically significant in estimating the nonlinear relationship between the time and the cefazolin concentration (*p*-values < 0.0001). There was also a significant effect of the presence of DH (*p*-value < 0.0001). The effect for DFDBA was not significantly different from no bone graft (*p*-value = 0.4308), nor was the effect for FDBA as compared to no bone graft (*p*-value = 0.3345). The results from the RCS model are presented in Figure 2.

**Figure 3:**
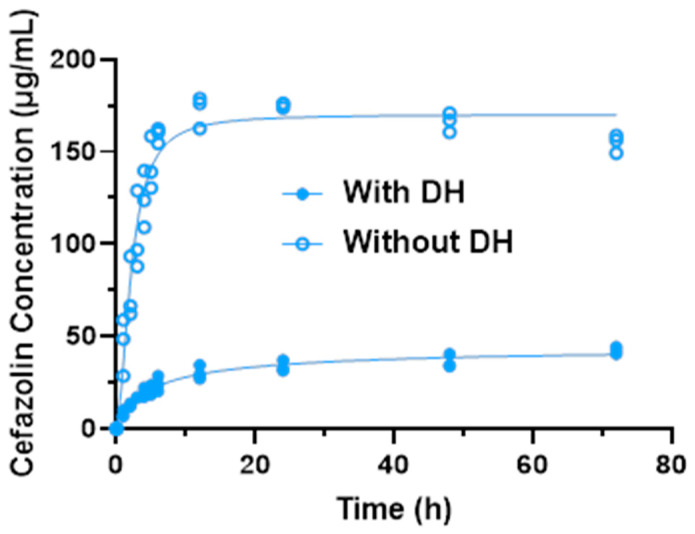
Logistic growth curves for cefazolin concentration with and without DH.

Due to the differences between the drug concentrations with and without DH, individual logistic growth models were fit for the two separately (Figure 3). For samples with DH (test), the estimated maximum concentration achieved was 36.97 ± 2.39 μg/mL (95% CI: 34.58–39.36). The estimated growth rate was 0.27 (95% CI: 0.17–0.37). At 4.17 h (95%: 3.26–5.07), the concentration reached half the maximum value. For samples without DH (control), the estimated maximum concentration achieved was 167.4 ± 7.0 μg/mL (95% CI: 160.4–174.4). The estimated growth rate was 0.70 (95% CI: 0.54–0.87). At 2.36 h (95% CI: 2.05–2.67), the concentration reached half the maximum value. These results demonstrate that with DH, the cefazolin concentration increased at a much slower and steady rate than the controls (without DH) (Table 1).

**Figure 4:**
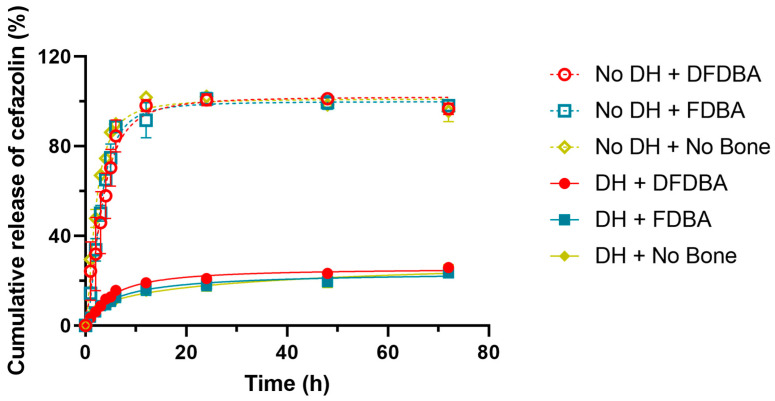
Cumulative release of cefazolin. The curve indicates that without DH, cephazolin is released fast and early, while all formulations containing DH demonstrate a sustained steady release.

Figure 4 shows the cumulative percent release of cefazolin from each sample over the course of the experiment, with 100% being calculated as 5 mg cefazolin within a 30 mL solution (100% release = 166.67 µg/mL). This figure shows that all of the samples without hydrogel showed a burst release of the cefazolin into solution, while approximately 75% of the cefazolin was retained within the samples containing hydrogel, even at the conclusion of the experiment at 72 h.

**Figure 5:**
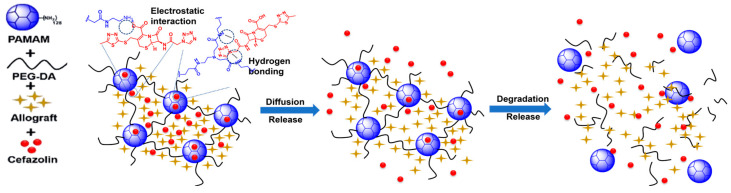
Schematic illustration of proposed mechanism of cefazolin binding/release from DH.

In this investigation, we were able to show the effectiveness of PAMAM/PEG-DA dendrimer hydrogels (DH) in slowing the in vitro release of cefazolin. The mechanism of this effect could be due to one or more of the following interactions between cefazolin and the DH (Figure 5):

1. Entrapment: many drug molecules can become entrapped within the network of the crosslinked DH.
2. Electrostatic interactions: positively charged unreacted amine groups on the surface of the dendrimers could have interacted with the negatively charged cefazolin carboxyl groups.
3. Hydrogen bonding between the cefazolin and the hydrogel network.

There is a clear dichotomy between entrapment and electrostatic interactions in this scenario, as any free amine group that is used to react with PEG-DA to facilitate entrapment of the drug will negate an amine group that could participate in electrostatic interactions. However, as stated before, the number of free amine groups should be minimized to limit the cytotoxicity of the DH, so it would be prudent to favor entrapment and hydrogen bonding, when possible, as the drivers of cefazolin–DH interactions. No chemical reaction is completely efficient; hence, there will always be some unreacted amine groups in the formulation. The entrapped drugs may release over a longer period with the gradual degradation of DH, which may facilitate the antibacterial effect for longer time.

One unexpected result from this experiment was the finding that the control group with neither DH nor bone graft did not show a consistent solution concentration. This may have been due to the use of the dialysis bag in the experiment. The objective of the dialysis bag was to simplify the sample analysis by preventing large molecular weight DH degradation products from exiting the dialysis bag. As a byproduct however, this may have slowed the diffusion of cefazolin outside of the dialysis bag, where it could be sampled. This suggests that all groups likely had a faster release profile than was observed in our experiment, as a dialysis bag was used in all groups.

It was hypothesized that the presence of bone graft material would delay the release of cefazolin, as cefazolin might bind to the mineralized portions of the graft. No evidence of such an interaction was found in this investigation; however, this does not rule out the possibility that such an interaction exists given the small sample size.

One limitation of this study was that we did not test the cytotoxicity of the DH to osteoblasts. Although previous studies have examined the effect of DH on other human cell lines [26,27], it will be important to examine their effect on osteoblasts if they are intended to be used in bone grafting procedures to be sure that they do not impede bone formation. Another limitation of this study was that the drug release was only monitored for 72 h, which is not long enough to observe the release of drugs entrapped in DH.

Future experiments may focus on the cytotoxicity of the cefazolin/DH drug delivery system via cell culture with osteoblasts, the drug release over a long period, or in vivo experiments to investigate the biocompatibility, the potential for antibiotic resistance with prolonged low-level release, and the effects on bone regeneration kinetics. Alternatively, studies concerning the delayed release of growth factors may be an interesting area of research, as such therapies are currently receiving much attention [28].

## 3. Conclusions

This study demonstrates the potential of PAMAM/PEG-DA dendrimer hydrogels (DH) for the sustained release of cefazolin in bone grafting procedures. The DH system significantly slowed the cefazolin release, retaining approximately 75% of the drug at 72 h compared to the rapid burst release in samples without DH. This controlled release could help prevent infections by maintaining effective antibiotic levels in graft sites where delayed angiogenesis reduces the efficacy of systemic antibiotics. While no significant interaction was found between cefazolin and the bone graft materials (FDBA and DFDBA), the DH effectively modulated the drug release through mechanisms such as entrapment and electrostatic interactions. The study’s limitations include the short term of the drug release experiments and the absence of cytotoxicity data for osteoblasts. Future research should focus on longer drug release periods, cytotoxicity assessments, and in vivo studies to confirm the biocompatibility and effectiveness in bone regeneration. This work provides a foundation for DH systems in both antibiotic delivery and broader applications such as growth factor release in regenerative medicine.

## 4. Materials and Methods

Freeze-dried bone allografts (FDBA) and demineralized freeze-dried bone allografts (DFDBA), purchased from LifeNet Health, Richmond, VA, USA, were suspended at a volume of 0.1 mL of allograft. Cefazolin solution was prepared from powder (Sigma) dissolved in pH 7.4 PBS at room temperature to a final concentration of 5 mg/mL Dendrimer hydrogel test group solutions were prepared using EDA-core PAMAM dendrimer generation 5 (G5) purchased from Dendritech (Midland, MI, USA) at a concentration of 10 wt. % G5. Polyethylene glycol diacrylate (PEG-DA, Mn = 575 g/mol) was then added to the test solutions in a 1:1 amine: acrylate molar ratio, immediately mixed using a vortex mixer at 3200 rpm, and left to solidify overnight at room temperature on an orbital shaker at 100 rpm.

- The experiment was set up to have 6 formulation groups:
- DFDBA with cephazolin (No DH + DFDBA);
- FDBA with cephazolin (No DH + FDBA);
- Free cephazolin (No DH + No Bone);
- DH with DFDBA with cephazolin (DH + DFDBA);
- DH with FDBA with cephazolin (DH + FDBA);
- DH with no bone (DH + No Bone).

After 24 h, all groups were sealed in dialysis bags along with 7.4 pH PBS pre-heated to 37 °C for a total of 30 mL of PBS in each group. The groups were then kept in a 37 °C bath for the duration of the experiment. One mL aliquots were sampled and replaced with 1 mL pre-heated PBS at the following time points: 1 h, 2 h, 3 h, 4 h, 5 h, 6 h, 12 h, 24 h, 48 h, 72 h. The aliquots were stored at 4 °C until analysis. The reverse-phase high performance liquid chromatography (RP-HPLC) system (Waters, Milford, MA, USA) consisting of a system Waters 1515 isocratic HPLC pump, a model Waters 717 plus autosampler, and a model Waters 2487 dual λ absorbance detector was used for the quantification of cefazolin. An XTerra particle-based RP-HPLC column (length 150 mm, particle size 5 μm, RP18) was purchased from Waters (MA, USA). An isocratic method was used, and the mobile phase consisted of ultrapure water and acetonitrile with 60:40 *v*/*v*, with the pH adjusted to 8 with triethylamine. The flow rate was set to 1 mL/min, using UV detection at 270 nm. The solutions were filtered through a 0.22 µm filter before being injected, and the mobile phase was degassed via ultrasonic bath before being used. The peak areas were integrated automatically, and the standard curve was developed using samples of cefazolin solubilized in PBS at various concentrations. All tests were run in triplicate.

**Statistics Methods**

A restricted cubic spline model was utilized to model the nonlinear cefazolin concentration by time and test for differences based on the presence of DH, FDBA, and DFDBA. Following the methodology established by Harrel [29], percentiles were used to estimate 4 knots in the concentration curves (5, 35, 65, 95th percentiles). Logistic growth models were used to estimate the maximum concentration, the rate of increase, and the time at which the concentration reached 50% of the maximum for the final models. SAS EG v.8.2 with SAS v9.4 were used for all analyses (SAS Institute, Cary, NC, USA). The significance level was preset at 0.05.

## Figures and Tables

**Table 1:** Key parameters for logistic growth curves for cefazolin concentration with and without DH.

Formulations	Maximum Concentration (μg/mL)	Growth Rate	Time to 50% Release (h)
With DH	36.97 ± 2.39	0.27	4.17
Without DH	167.4 ± 7.0	0.70	2.36

## Data Availability

The original contributions presented in the study are included in the article, further inquiries can be directed to the corresponding author/s.
